# mLST8 Promotes mTOR-Mediated Tumor Progression

**DOI:** 10.1371/journal.pone.0119015

**Published:** 2015-04-23

**Authors:** Kyoko Kakumoto, Jun-ichiro Ikeda, Masato Okada, Eiichi Morii, Chitose Oneyama

**Affiliations:** 1 Department of Oncogene Research, Research Institute for Microbial Disease, Osaka, University, Suita, Osaka, Japan; 2 Department of Pathology, Graduate School of Medicine, Osaka University, Suita, Osaka, Japan; Roswell Park Cancer Institute, UNITED STATES

## Abstract

The activity of the mechanistic target of rapamycin (mTOR) is elevated in various types of human cancers, implicating a role in tumor progression. However, the molecular mechanisms underlying mTOR upregulation remain unclear. In this study, we found that the expression of mLST8, a required subunit of both mTOR complex 1 (mTORC1) and complex 2 (mTORC2), was upregulated in several human colon and prostate cancer cell lines and tissues. Knockdown of mLST8 significantly suppressed mTORC1 and mTORC2 complex formation, and it also inhibited tumor growth and invasiveness in human colon carcinoma (HCT116) and prostate cancer (LNCaP) cells. Overexpression of mLST8 induced anchorage-independent cell growth in normal epithelial cells (HaCaT), although mLST8 knockdown had no effect on normal cell growth. mLST8 knockdown reduced mTORC2-mediated phosphorylation of AKT in both cancer and normal cells, whereas it potently inhibited mTORC1-mediated phosphorylation of 4E-BP1 specifically in cancer cells. These results suggest that mLST8 plays distinct roles in normal and cancer cells, depending upon its expression level, and that mLST8 upregulation may contribute to tumor progression by constitutively activating both the mTORC1 and mTORC2 pathways.

## Introduction

The mammalian target of rapamycin (mTOR) is a serine/threonine kinase belonging to the phosphatidylinositol 3-kinase (PI3K)-related kinase family. mTOR assembles into two large protein complexes, mTOR complex 1 (mTORC1) and mTOR complex 2 (mTORC2), which are independently regulated by distinct binding partners [1–4]. mTORC1 specifically contains RAPTOR and PRAS40, while mTORC2 contains RICTOR, mSIN1, and PROTOR. mTORC1 is activated by diverse stimuli such as nutrients and growth factors and also by the PI3K–AKT pathway, and the complex controls cell growth by regulating protein synthesis via phosphorylation of its downstream substrates, ribosomal S6 kinase (S6K) and 4E-binding protein 1 (4E-BP1) [5, 6]. By contrast, mTORC2 regulates cell proliferation, survival, and the actin cytoskeleton by activating AKT, protein kinase C-α (PKC-α), and serum- and glucocorticoid-induced protein kinase 1 (SGK-1) [7, 8]. Both mTOR pathways are frequently deregulated in human cancers. Recent work has shown that various cancer cells have elevated mTOR activity due to upregulation of mTOR complex components, e.g., mTOR, RICTOR, RAPTOR, mSIN1, PRAS40, and DEPTOR [9–14]. Mutation of PTEN, a negative regulator of the PI3K–AKT pathway, has also been implicated in activation of mTORC1 signaling in cancers [15]. Furthermore, mutation of mTOR itself contributes to the risk of various cancers [16–19]. Based on these findings, the mTOR pathway is regarded as a promising therapeutic target for some human cancers; consequently, specific inhibitors of mTOR complexes, such as rapamycin analogs (Rapalogs) and mTOR kinase inhibitors, are being actively developed [20, 21].

The molecular mechanisms underlying regulation of mTOR activity have been elucidated by a co-crystal structure of a complex of mTOR and mammalian lethal with SEC13 protein 8 (mLST8), also known as GbetaL [22]. mLST8 is a common subunit of both mTORC1 and mTORC2, and is necessary for activation of the mTOR kinase [23]. The structure of the mTOR–mLST8 complex revealed that mLST8 directly stabilizes the active site of mTOR, supporting the idea that mLST8 plays a critical role in mTOR kinase activity. Analyses of mLST8-knockout mouse embryos and fibroblasts have shown that mLST8 is required for formation of mTORC2, suggesting a specific role for mLST8 in mTORC2 function as well [24]. Furthermore, mLST8 can associate with other cellular proteins, such as CAD, a multifunctional protein involved in pyrimidine synthesis, which is phosphorylated by S6K [25, 26]. Thus, mLST8 is critical for the proper regulation of mTOR pathways, but its precise function still needs to be defined. Also, the contribution of mLST8 to carcinogenesis and/or progression of human cancers, particularly those in which mTOR pathways are deregulated, remains uncharacterized.

We previously found that expression levels of certain components of mTOR complexes, such as mTOR itself and RICTOR, are upregulated in various human cancers as a result of silencing of specific microRNAs [9, 10]. In this study, we show that mLST8 is also upregulated in several human colon and prostate cancer cells/tissues, in which it contributes to tumor growth and invasion. Upregulated mLST8 is required for activation and assembly of both mTORC1 and mTORC2 in cancer cells, although perturbation of mLST8 does not affect proliferation of normal cells. Our results suggest that mLST8 plays distinct roles in normal and cancer cells, depending on its expression level, and that upregulation of mLST8 contributes to tumor progression by activating both the mTORC1 and mTORC2 pathways.

## Results

### Expression of mLST8 is upregulated in various colon and prostate tumors

**Fig 1:**
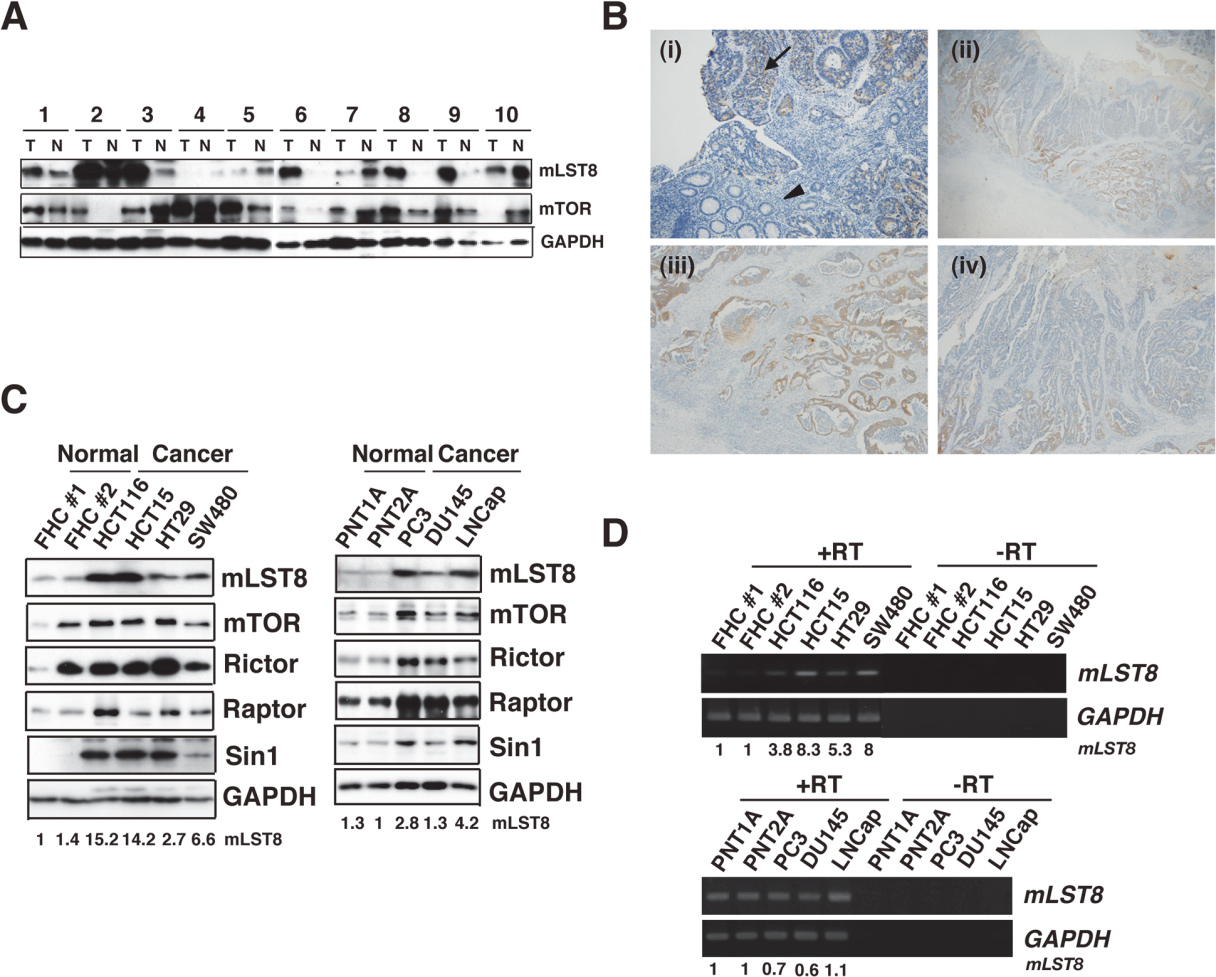
Expression of mLST8 is upregulated in human colon and prostate cancer. (A) Western blot analysis with the indicated antibodies. Total cell lysates from normal tissues and tumor tissues from human colorectal cancer patients. (B) (i) Immunohistochemistry for mLST8 (×100) in human colorectal adenocarcinoma (arrow) and non-cancerous (arrowheads) tissue samples. Moderate and high intensities of mLST8 were observed in 16 out of 20 samples, and representative images from these 16 samples are shown. (ii) In samples positive for mLST8, strong expression was observed in invasive cancer lesions (×12.5). (iii) Invasive tumor cell nests exhibited strong expression of mLST8 in (ii) (×40), but no mLST8 expression in surface lesions (iv; ×40). (C) Western-blot analysis of the indicated antibodies. Total cell lysates from various types of human normal colorectal and colon-cancer cells (left panels) and human normal prostate and prostate-cancer cells (right panels). (D) Expression of *mLST8* mRNA was analyzed by RT-PCR. Samples were prepared from several human colon (upper panels) and prostate cell lines (lower panels).

To examine the functional relevance of mLST8 to human cancers, we first analyzed expression levels of mLST8 protein in human colorectal primary tumors. Western-blot analyses revealed that mLST8 had a tendency to be upregulatied in five out of ten cancerous tissues relative to the levels in normal tissues (Fig 1A). We also observed that mLST8 upregulation was associated with that of mTOR. The intensity of mLST8 immunoreactivity was also higher in cancerous lesions than in normal tissues in 16 out of 20 samples examined [Fig 1B (i)]. In these clinical samples, the invading edges of tumors exhibited strong mLST8 expression [Fig 1B (ii)(iii)(iv)]. We next examined mLST8 expression in several lines of colon and prostate cancer cells (Fig 1C). Western-blot analysis revealed a marked upregulation of mLST8 in all cancer cell lines tested. Similarly, protein levels of other mTOR complex components, such as mTOR, RICTOR, RAPTOR, and mSIN1, were also upregulated in these cells. Furthermore, RT-PCR analysis of *mLST8* mRNA levels revealed that mLST8 expression is regulated at the level of transcription in colon cancers (Fig 1D, upper panels). In prostate cancers, however, there was no significant change in the levels of transcripts between cancer and normal cells, suggesting that mLST8 expression may instead be regulated by protein stability in these cells (Fig 1D, lower panels). These results suggest that mLST8 is upregulated coordinately with mTOR complex components in some human tumors and cancer cell lines.

### mLST8 regulates tumor growth *in vitro and in vivo*

**Fig 2:**
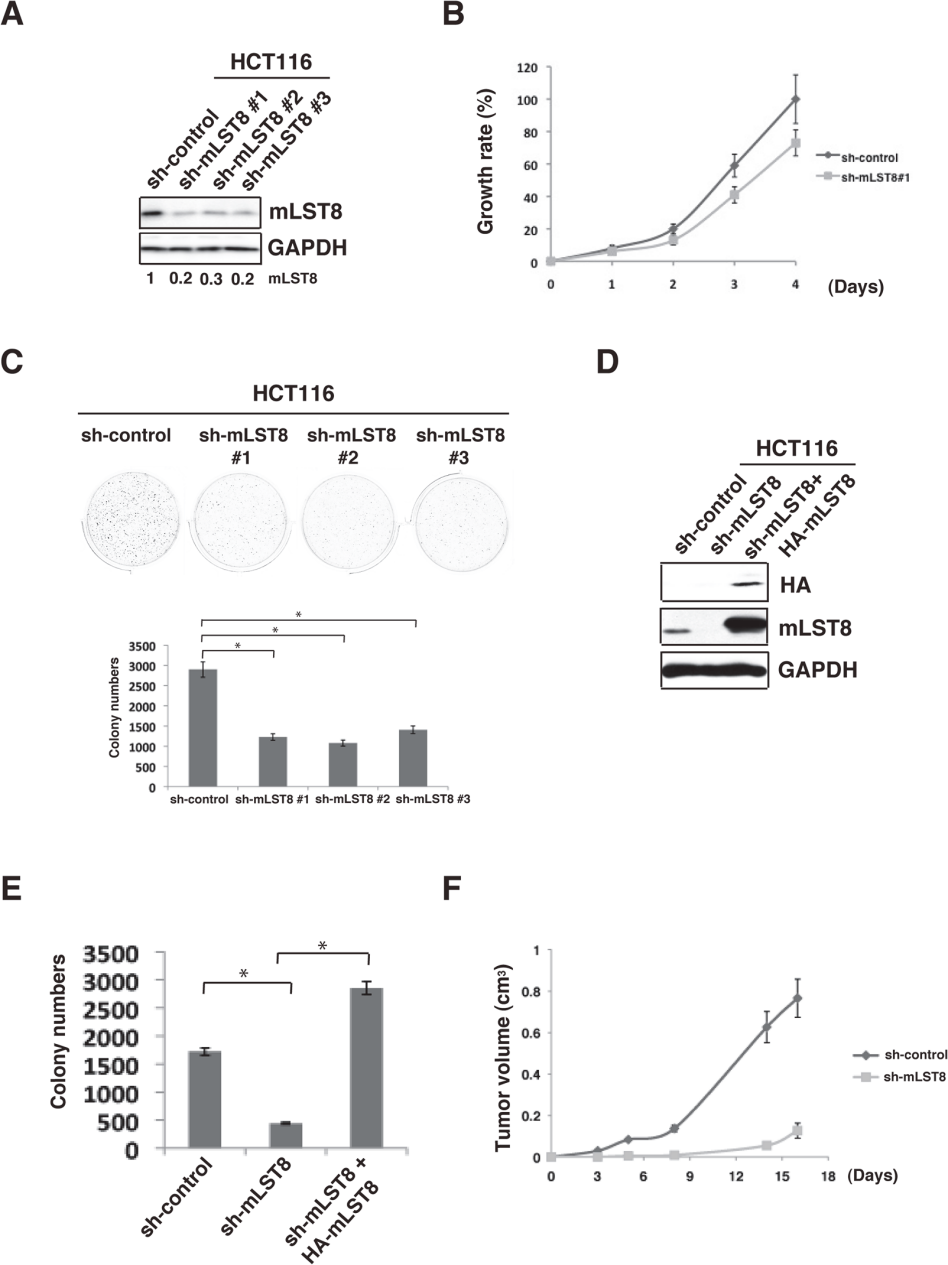
mLST8 promotes tumorigenesis. (A) Total cell lysates from HCT116 cells expressing control shRNA (sh-control) or mLST8 shRNA (sh-mLST8) were immunoblotted with the indicated antibodies. (B) Proliferation of HCT116 cells expressing control shRNA (sh-control) or mLST8 shRNA (sh-mLST8) was examined by an *in vitro* proliferation assay using WST-1. Mean values ± S.D. were obtained from three independent experiments. (C) Soft-agar colony-formation assay with HCT116 cells expressing control or mLST8 shRNA. Representative dishes obtained from three independent experiments are shown (upper panels). Quantitative graph shows colony numbers from three independent experiments (lower graph). (D) Total cell lysates from HCT116 cells expressing control shRNA, mLST8 shRNA, or mLST8 shRNA with HA-tagged mLST8 (sh-mLST8 + HA-mLST8) were immunoblotted with the indicated antibodies. (E) Soft-agar colony-formation assay with the cells indicated in (D) were performed over 7 days. Average numbers of colonies / cm^2^ ± S.D. are shown. (F) HCT116 cells expressing control (sh-control) or mLST8 shRNA (sh-mLST8) were inoculated subcutaneously into nude mice. Averages ± S.D. of tumor volumes (cm^3^) obtained from four to five mice are plotted versus days after inoculation. *, p < 0.05 by Student’s t test (C)(E).

**Fig 3:**
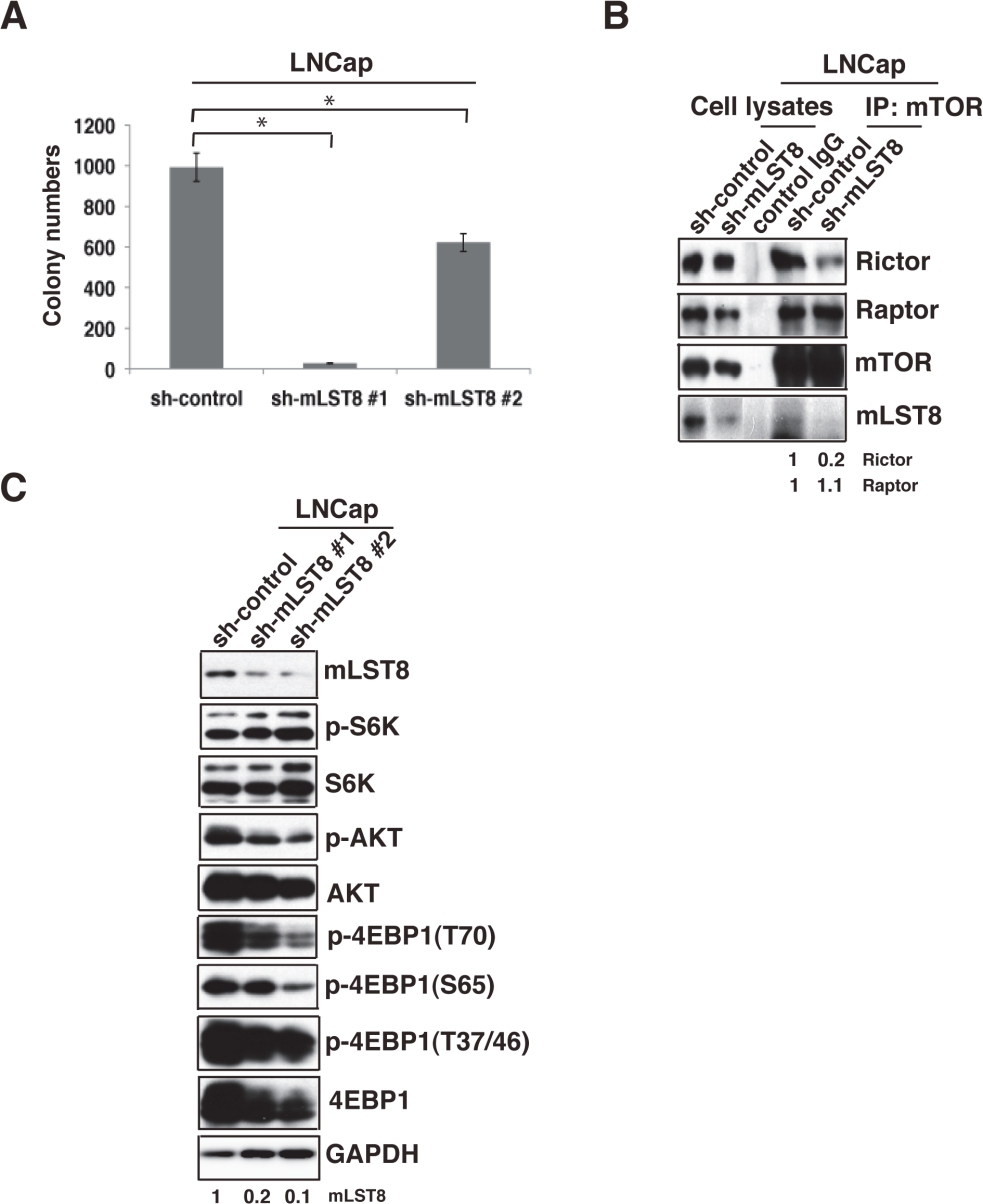
mLST8 promotes mTORC1/2-mediated transformation of prostate cancer cells. (A) LNCaP cells expressing control shRNA or mLST8 shRNA were subjected to soft-agar colony-formation assay over 7 days. The mean number of colonies ± S.D. was obtained from three independent experiments. *, p < 0.05 by Student’s t test. (B) Immunoprecipitation of mTOR complex from LNCaP cells expressing control shRNA (sh-control) or mLST8 shRNA (sh-mLST8). Western-blot analysis of total cell lysates and samples immunoprecipitated with the indicated antibodies. (C) Activation status of several mTOR substrates in LNCaP cells indicated in (A) was analyzed by immunoblotting with the indicated antibodies.

**Fig 4:**
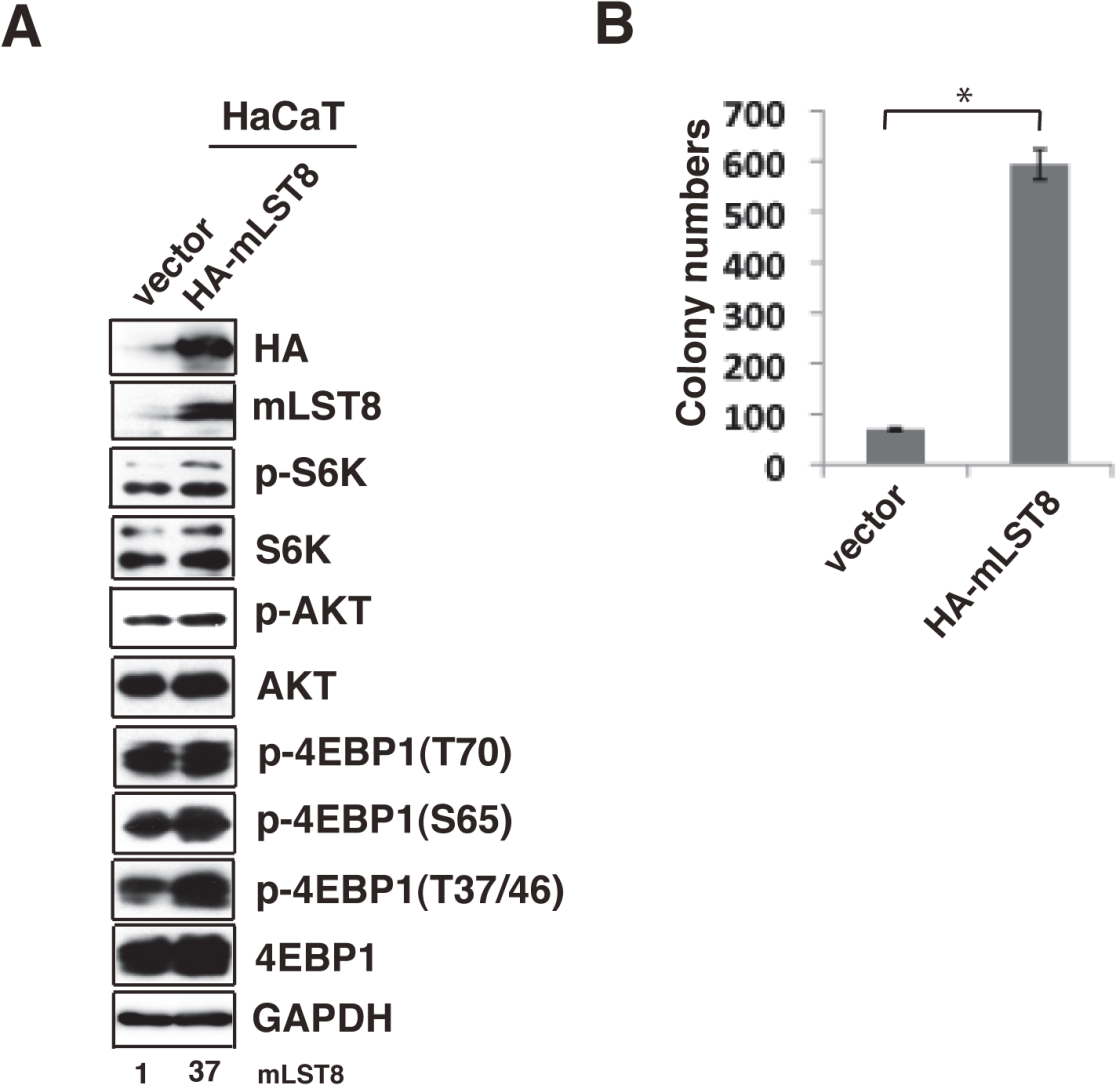
Overexpression of mLST8 promotes transformation. (A) Western-blot analysis, using the indicated antibodies, of HaCaT cells expressing empty vector or HA-tagged mLST8. (B) Soft-agar colony-formation assay with the cells indicated in (A), stained with MTT after 3 weeks. The graph shows MTT-positive colonies. *, p < 0.05 by Student’s t test.

To evaluate the role of mLST8 upregulation in cancer cells, we examined the effects of shRNA-mediated knockdown of mLST8 (mLST8-KD) on tumor growth of HCT116 (Fig 2) and LNCaP cells (Fig 3). mLST8-KD suppressed the growth rate of HCT116 cells (Fig 2A and 2B). More notably, compared to control KD, mLST8-KD induced a marked reduction in anchorage-independent growth of HCT116 cells and LNCaP cells (Figs 2C and 3A). Overexpression of HA-tagged mLST8 in mLST8-KD cells restored the ability of anchorage-independent growth to a level even higher than that of control cells (Fig 2D and 2E). Overexpression of mLST8 also promoted colony-forming activity in non-transformed human keratinocyte HaCaT cells, although the colony-forming activity in these cells was substantially weaker than those in cancer cells (Fig 4). Furthermore, the effect of mLST8-KD was evident *in vivo*: mLST8-KD in HCT116 cells potently suppressed tumorigenesis in nude mice (Fig 2F). These results suggest that the expression levels of mLST8 are tightly associated with the potential for tumor growth.

### mLST8 regulates mTORC1/2 activity in cancer cells

**Fig 5:**
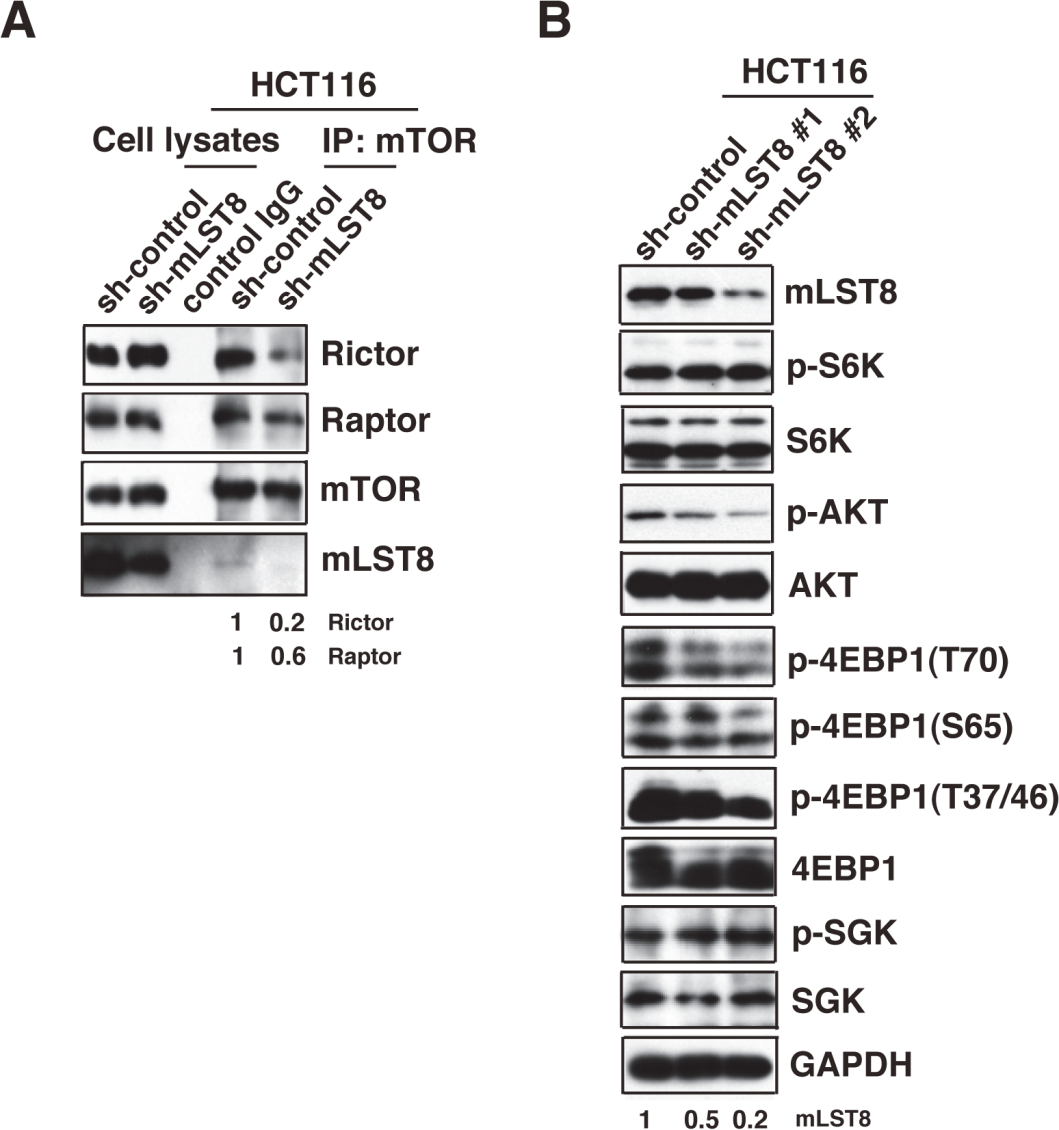
mLST8 regulates mTORC1/2 signaling pathways in cancer cells. (A) Immunoprecipitation of mTOR complex in HCT116 cells expressing control shRNA (sh-control) and mLST8 shRNA (sh-mLST8). Western-blot analysis of the total cell lysates and immunoprecipitated samples were performed with the indicated antibodies. The relative protein levels of Rictor or Raptor bound to mTOR are shown at the bottom of the panels. (B) Western-blot analysis of phosphorylation status of several mTOR substrates. Total cell lysates obtained from the cells indicated in Fig 2A were immunoblotted with the indicated antibodies.

To investigate the mechanism underlying mLST8-mediated promotion of tumor growth, we assessed the effects of mLST8-KD on the formation of mTOR complexes and the activity of downstream signaling components in HCT116 and LNCaP cells. Immunoprecipitation assays with anti-mTOR antibody revealed that mLST8-KD decreased the amounts of RICTOR and RAPTOR in mTOR complexes, with a larger reduction in RICTOR (Figs 3B and 5A). These results suggest that mLST8 is required for stable formation of both mTORC1 and mTORC2, although it preferentially contributes to mTORC2 formation. Western-blot analyses of the phosphorylation status of downstream components of mTOR pathways revealed that mLST8-KD induced significant reduction in phosphorylation of AKT and 4E-BP1 in both cell types, whereas the phosphorylation status of S6K and SGK1 was unchanged (Figs 3C and 5B). These data suggest that mLST8 upregulation contributes to promotion of mTORC1/2 formation and subsequent phosphorylation of AKT and 4E-BP1.

### mLST8 does not affect cell proliferation of normal cells

**Fig 6:**
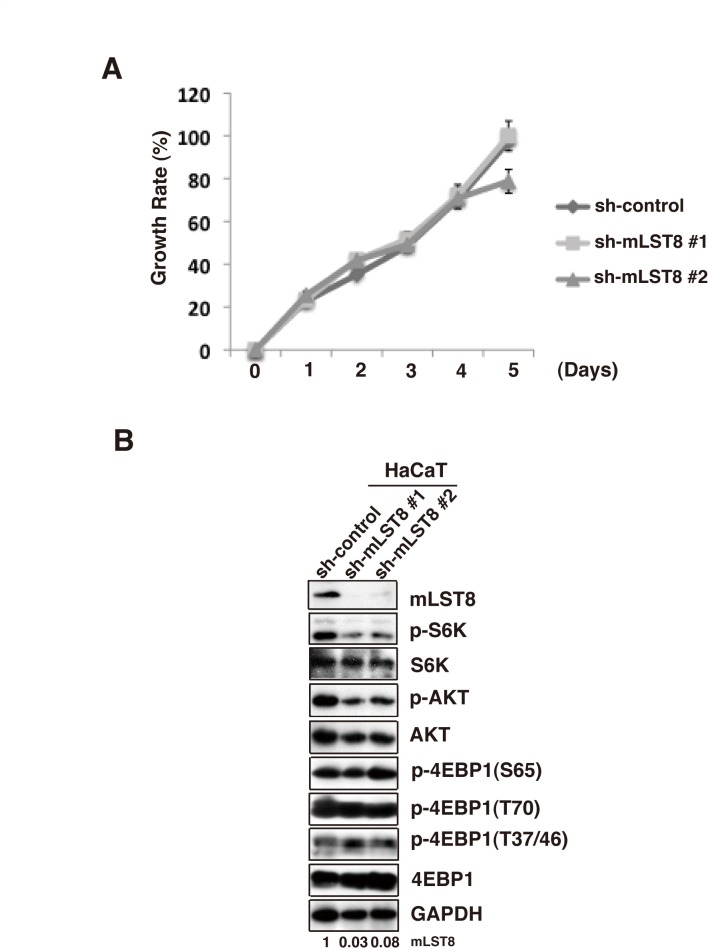
mLST8 is not required to cell proliferation of normal cells. (A) Proliferation of HaCaT cells expressing control shRNA (sh-control) or mLST8 shRNA (sh-mLST8) was examined by an *in vitro* proliferation assay using WST-1. Mean values ± S.D. were obtained from three independent experiments. (B) Western-blot analysis of whole-cell lysates from the cells indicated in (A) 2 days after plating.

We then examined the effect of mLST8-KD on the growth of normal and immortalized human keratinocytes (HaCaT). Although mLST8 was effectively knocked down in these cells (Fig 6B), mLST8 downregulation did not affect growth rate (Fig 6A). Under these conditions, phosphorylation of S6K and AKT was slightly decreased by mLST8-KD, whereas phosphorylation of 4E-BP1 was almost unchanged (Fig 6B). These observations suggest that perturbation of mLST8 is not crucial for growth of normal cells, and that the role of mLST8 in regulating mTOR pathways may differ between normal and cancer cells.

### mLST8 regulates invasiveness of cancer cells

**Fig 7:**
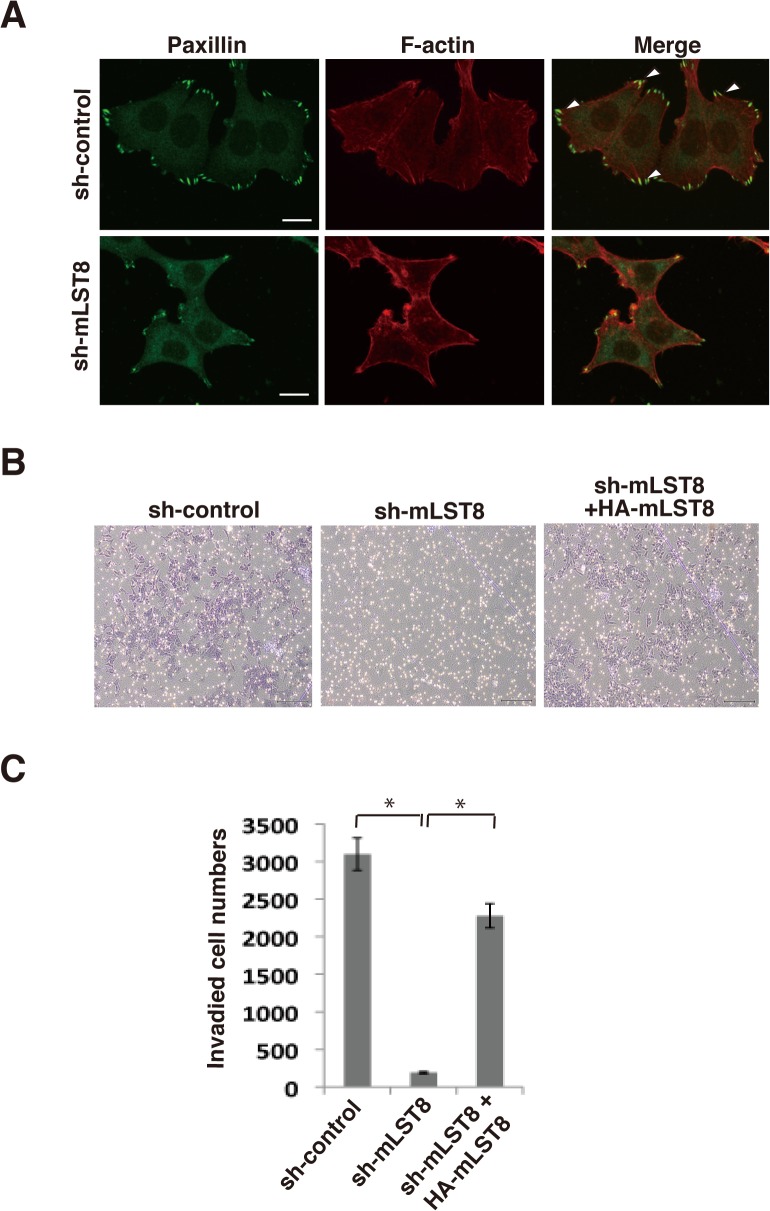
mLST8 regulates cell motility and invasiveness of human colon cancer cells. (A) HCT116 expressing control RNA (sh-control) and mLST8 shRNA (sh-mLST8) were subjected to immunocytochemistry. Paxillin (green) and F-actin (red) were analyzed by immunostaining of the indicated cells grown on fibronectin-coated dishes. Locations of focal contacts are indicated by arrowheads. Scale bar = 20 μm. (B) *In vitro* invasion assays of HCT116 cells expressing control shRNA, mLST8 shRNA, and mLST8 shRNA + HA-tagged mLST8. Cells were seeded into Matrigel invasion chambers. After 48 h, membranes were detached, and cells were stained and counted. (C) The mean numbers of invading cells shown in (B) (cells per cm^2^ ± S.D.) were obtained from three independent experiments. *, p < 0.05 by Student’s t test.

In addition to exerting effects on tumor growth, mLST8-KD also induced dramatic morphological changes in HCT116 cells. Cell staining for actin fibers (F-actin) and paxillin, a marker of focal contact, showed that mLST8-KD caused disruption of stress fibers and attenuated formation of focal contacts (Fig 7A). Previously, similar effects were observed by knockdown of Rictor in these cells [10]. Taken together with these observations, this result suggests that mLST8 upregulation promotes cytoskeletal reorganization and formation of focal contacts, potentially through activation of the mTORC2 pathway. Because the ability to form focal contacts is functionally linked to invasiveness of cancer cells, we examined the effect of mLST8-KD on *in vitro* invasive activity of HCT116 cells using a Matrigel-based chamber assay. mLST8-KD strongly suppressed the invasive activity of HCT116 cells, and the expression of HA-tagged mLST8 in mLST8-KD cells restored their invasive activity (Fig 7B and 7C). These findings suggest that mLST8 upregulation increases the invasive potential of cancer cells.

## Discussion

In this study, we addressed the role of mLST8, a requisite component of mTOR complexes, in tumor progression. We found that mLST8 is upregulated in some human cancer tissues and cells, and that upregulated mLST8 promotes mTORC1/2 formation and induces activation of AKT and phosphorylation of 4E-BP1, resulting in promotion of tumor growth as well as invasive potential of cancer cells.

Our findings provide the first evidence that mLST8 is upregulated in a subset of human cancers; however, the molecular mechanisms underlying mLST8 upregulation are currently unknown. In colon cancers, the levels of mLST8 transcripts were elevated, suggesting that activation of some transcription factors and/or silencing of particular microRNAs may be involved in mLST8 upregulation, as observed for other components of the mTOR complexes [9, 10, 27–31]. In the case of prostate cancers, however, there was no significant change in the expression of mLST8 transcripts between normal and cancer cells. Previous studies suggested that upregulation of some components of mTORC1 and mTORC2 activates mTOR signaling, potentially due to mutual stabilization between components of mTORC1 and mTORC2[32]. We also observed that mLST8 upregulation was associated with upregulation of other components of mTOR complexes, such as mTOR itself and RICTOR/RAPTOR. Therefore, it is possible that mLST8 upregulation can be attributed to protein stabilization, which is in turn caused by upregulation of binding partners that are upregulated by other mechanisms, e.g., by microRNA silencing [9]. On the other hand, several mutations in the *mLST8* gene have recently been identified in cancer patients based on information in the Cosmic and CBio cancer genome databases [33]. The potential contributions of these mutations to mLST8 upregulation also deserve further investigation.

Despite the potential importance of mLST8 in regulating mTOR signaling, knockdown of mLST8 had no effect on the growth of normal epithelial cells. Also, mLST8 had little effect on phosphorylation of S6K and AKT in these cells (Fig 6). Ablation of mLST8 in mice has revealed that mLST8 is not essential for normal development [24]. These observations suggest that mLST8 plays a dispensable role in regulating mTOR function under normal conditions. By contrast, mLST8 knockdown in cancer cells led to reduction in formation of mTORC1/2 and phosphorylation of AKT and 4E-BP1, consistent with the results of a previous study (Figs 3 and 5, [24]). While the reason for the difference of phosphorylation between S6K and 4E-BP1 as mTORC1 substrate is unclear, it may relate to an independent pathway of regulation of S6K1 and 4E-BP in cancer cells [34]. We also observed that overexpression of mLST8 induces colony-forming activity in non-transformed HaCaT cells, accompanied by a slight increase in phosphorylation of S6K, AKT, and 4E-BP1 (Fig 4). Therefore, it is likely that upregulation of mLST8 induces full activation of mTORC1/2, resulting in activation of the downstream substrates that are required for tumor progression. It is well known that phosphorylation of 4E-BP1 is responsible for tumor progression in various types of cancer [35]. Upregulated mLST8 induces phosphorylation of 4E-BP1, thereby stimulating cap-dependent translation of genes involved in cell growth and ultimately promoting of tumor progression. In this context, mLST8 may play distinct roles in normal and cancer cells, depending upon its expression levels.

Recent analysis of the mTOR–mLST8 complex structure revealed that the binding of mLST8 contributes to stabilization of the active site of mTOR [22]. In this study, we showed that mLST8 knockdown induces dissociation of mTORC1/2 complexes in cancer cells. Therefore, it is possible that upregulation of mLST8 increases the population of fully active and stable mTOR complexes, whereas reduction in mLST8 levels induces structural change in mTOR and affects its binding affinity for other components such as RAPTOR and RICTOR, as well as its substrates. In fact, affinity for 4E-BP1 is dramatically reduced when RAPTOR is absent from mTORC1 [22, 36]. This notion is further supported by recent studies demonstrating that phosphorylated RAPTOR and SIN1 promote binding between components of the mTOR complex and its substrates [33, 37]. Because mLST8 is a required subunit of mTOR kinase, dissociation of mLST8 from mTOR complex may induce critical changes in the catalytic activity and substrate specificity of mTOR complexes. The distinct functions of mTOR in cancer and normal cells may be due to structural changes in mTOR complexes that are dependent on mLST8 levels.

In conclusion, we have demonstrated a crucial role for mLST8-mediated upregulation of the mTOR pathway in promoting tumor progression. Our study provides new insights into the regulatory mechanisms of the mTOR signaling pathway. Further analyses of mLST8-mediated regulation of mTOR pathways may provide new targets for therapeutic intervention in a wide variety of human cancers.

## Material and Methods

### Cells and materials

A human keratinocyte cell line (HaCaT), human colon cancer cells (HCT116, HCT15, HT29 and SW480), two different types of FHC (normal human colon cells), human prostate cancer cells (PC3, DU145, and LNCaP), and normal human prostate cells (PNT1A and PNT2A) were obtained from the American Type Culture Collection (ATCC). PC3, HCT15, and HT29 cells were cultured in Dulbecco’s modified Eagle’s medium (DMEM). PNT1A, PNT2A, LNCaP, and DU145 cells were cultured in RPMI medium. HCT116 cells were cultured in McCoy’s 5A medium. All media were supplemented with 10% fetal bovine serum (FBS). FHC cells were cultured in DMEM/Ham’s F-12 (1:1) with 10% FBS, 5 μg/ml insulin, 5 μg/ml transferrin, 100 ng/ml hydrocortisone, and α-MEM (minimal essential medium) with 10% FBS. Frozen colon tissues were divided into tumor (T) and non-cancerous (N) regions as defined by two pathologists (JI and EM). The research protocol for the collection of human samples was approved by the ethical review board of the Graduate School of Medicine Osaka University, Japan. Informed consent was obtained from all patients in writing before enrollment in the study.

### Growth assay

Cells were seeded at 5 × 10^2^ cells/well in 96-well plates, and then incubated for the indicated times. At the end of time points, cells were incubated with 10 μl per well of WST-1 assay reagent (Roche). After 30-min incubation, absorbance was measured at 450 nm. Each experiment was repeated three times.

### Immunohistochemistry

Histologic specimens were fixed in 10% formalin and processed routinely for paraffin embedding. Histological sections (4 μm thick) were stained with hematoxylin and eosin and reviewed by two pathologists (JI and EM) to define cancerous and corresponding normal tissues. An immunoperoxidase procedure was performed on the paraffin-embedded sections, as described previously [9]. After antigen retrieval using a Pascal pressurized heating chamber (Dako A/S, Glostrup, Denmark), the sections were incubated with anti-mLST8 antibody diluted 1:50. Cells were then treated with a ChemMate EnVision kit (Dako). Diaminobenzidine (Dako) was used as the chromogen. As a negative control, staining was carried out in the absence of primary antibody. Stained sections were evaluated independently by two pathologists (JI and EM).

### Immunochemical analysis

Cells were lysed in 2× SDS sample buffer containing 1.25 mM Tris-HCl (pH 6.8), 0.4% SDS, 20% glycerol, and 1 mM Na_3_VO_4_, and then boiled for 10 min. An aliquot of each lysate (5–10 μg protein) was separated on 8–14% SDS-PAGE gel and transferred to polyvinylidene difluoride (PVDF) membranes (Millipore). The following antibodies were used: anti-4EBP1, anti–phospho-4EBP1 (S37/T46), anti–phospho-4EBP1 (S65), anti–phospho-4EBP1 (T70), anti-AKT, anti–phospho-AKT (S478), anti-S6K, anti–phospho-S6K (T308), anti-mTOR, anti-RAPTOR, anti-RICTOR, anti-p-SGK (S422), and SGK antibodies were from Cell Signaling Technology; anti-Sin1 and anti-mLST8 antibodies were from Abcam; anti-GAPDH antibody was from Santa Cruz Biotechnology; and anti-PTEN antibody was from CASCADE. For immunocytochemistry, performed as described previously, the following antibodies were used: mouse monoclonal anti-paxillin (BD Bioscience) and Alexa Fluor 488–conjugated goat anti-mouse IgG (Molecular Probes). Alexa Fluor 594–conjugated phalloidin was obtained from Molecular Probes.

### Immunoprecipitation

Cells were lysed in ice-cold lysis buffer [40 mM HEPES (pH 7.5), 120 mM NaCl, 1 mM EDTA, 10 mM pyrophosphate, 10 mM glycerophosphate, 50 mM NaF, 0.3% CHAPS] containing protease cocktail (Nacalai Tesque). After clearing the lysate by centrifugation at 13,000 × g for 10 min, 4 μg of immunoprecipitating antibody was added to the supernatant. After 1.5-hr incubation at 4°C, 30 μl of 50% slurry of protein G–Sepharose was added, and the mixture was incubated for 1 hr at 4°C, after which immunoprecipitates were washed four times with lysis buffer. Samples were resolved by SDS-PAGE, and proteins were transferred to PVDF and subjected to immunoblotting as described above.

### Invasion assay

Invasion assays were performed as described [38]. Briefly, a suspension of 5 × 10^4^ cells in serum-free medium was loaded into the upper well of BioCoat Matrigel Invasion Chambers (BD Science), and conditioned medium from NIH3T3 cells (as a chemoattractant) was loaded in the lower well. After 48-hr incubation, non-invaded cells were removed with a cotton swab, and migrated cells were fixed with methanol, stained with toluidine blue, and counted.

### Retroviral-mediated gene transfer

Retroviral gene transfer was performed as described previously [39]. Full-length human *mLST8* cDNA was amplified by PCR, and then subcloned into pCX4bsr-HA-tagged (N-terminus). An *mLST8* allele that escapes targeting by sh-mLST8 was generated by QuikChange mutagenesis method using the following primers: 5’-gaacctgcagtgccaacgaatatttcaagtaaatgcacccattaactgc-3’ and 5’-gcagttaatgggtgcatttacttgaaatattcgttggcactgcaggttc -3’.

### Lentiviral-mediated sh-RNA

Control vector and vector carrying human *mLST8* (ID: TRCN0000039759, TRCN0000039761 and TRCN0000039762) were purchased from Sigma–Aldrich. Production of purified lentivirus was performed according to the manufacturer’s instructions. The stock cells after preparation were subjected to each experiment.

### RT-PCR

Total RNA was extracted from normal and carcinoma cells, including colorectal and prostate cells, using the Sepasol reagent (Nacalai Tesque). After reverse transcription, PCR was carried out using primer sets for human *mLST8* (5’-tgattgctgctgcaggttac-3’ and,5’- gttaatgggtgcgttcacct-3’) and human *GAPDH* (5’-accagaagactgtggatgg-3’ and 5’-tgctgtagaaaaattcgttg -3’).

### Soft-agar colony formation assay

Single-cell suspensions were plated in 6-well culture dishes (1 × 10^4^ cells per well in 1.5 ml of DMEM containing 10% FBS and 0.36% agar, on a layer of 2.5 ml of the same medium containing 0.7% agar). Seven days after plating, colonies were stained with 3-(4,5-dimethylthiazol-2-yl)-2,5-diphenyltetrazolium bromide (MTT); photographs of the stained colonies were taken; and the colonies were counted.

### Tumorigenesis assays

Immunodeficient mice (BALB/c Slc-*nu/nu*, Japan SLC, Inc.) were subcutaneously injected with 1 × 10^6^ cells suspended in 200 μl of serum-free McCoy’s 5A for one location. Tumors were monitored every 2 or 3 days, and then mice and tumor volumes were calculated using the following formula: 0.5 × *L* × W^2^. The mice were sacrificed after 16 days with tumor volume less than 1 cm^3^ of monitoring. The mice used this study were housed in environmentally-controlled rooms of the animal experimentation facility at Osaka University and sacrificed under deep anesthesia by inhaling 4% isoflurane. Experiments were conducted under the applicable laws and guidelines for the care and use of laboratory animals in the Research Institute for Microbial Diseases, Osaka University, approved by the Animal Experiment Committee of the Research Institute for Microbial Disease, Osaka University.
